# A Phase 1/2a Study Evaluating Safety and Immunogenicity of Ad26.RSV.preF in RSV-seronegative Toddlers Aged 12–24 Months

**DOI:** 10.1093/ofid/ofae453

**Published:** 2024-08-08

**Authors:** Joanne M Langley, Terry M Nolan, Mika Rämet, Peter C Richmond, Nelson Rosário Filho, Wouter Haazen, Sara P H van den Berg, Kristi Williams, Arangassery Rosemary Bastian, Edmund Omoruyi, Joanna Williams Durkin, Nadine Salisch, Gunter Van Geet, Wilbert van Duijnhoven, Esther Heijnen, Benoit Callendret

**Affiliations:** Canadian Center for Vaccinology, Dalhousie University, IWK and Nova Scotia Health, Halifax, Nova Scotia, Canada; Department of Infectious Diseases, Peter Doherty Institute for Infection and Immunity at The University of Melbourne, Melbourne, Victoria, Australia; Murdoch Children's Research Institute, Melbourne, Victoria, Australia; FVR – Finnish Vaccine Research Ltd., and Faculty of Medicine and Health Technology, Tampere University, Tampere, Finland; Wesfarmers Centre of Vaccines and Infectious Diseases, Telethon Kids Institute, University of Western Australia, Perth, Western Australia, Australia; Discipline of Paediatrics, School of Medicine, University of Western Australia, Perth, Western Australia, Australia; Division of Allergy and Immunology, Complexo Hospital de Clínicas da Universidade Federal do Paraná, Curitiba, Brazil; Janssen Vaccines & Prevention B.V., Leiden, The Netherlands; Janssen Vaccines & Prevention B.V., Leiden, The Netherlands; Janssen Vaccines & Prevention B.V., Leiden, The Netherlands; Janssen Vaccines & Prevention B.V., Leiden, The Netherlands; Janssen Infectious Diseases, Beerse, Belgium; Janssen Biologics Europe, Leiden, The Netherlands; Janssen Vaccines & Prevention B.V., Leiden, The Netherlands; Janssen Research & Development, Beerse, Belgium; Janssen Research & Development, Beerse, Belgium; Janssen Vaccines & Prevention B.V., Leiden, The Netherlands; Janssen Vaccines & Prevention B.V., Leiden, The Netherlands

**Keywords:** adenovirus serotype 26, respiratory syncytial virus, pediatric vaccination, respiratory syncytial virus vaccine

## Abstract

**Background:**

Respiratory syncytial virus (RSV) causes serious illness in children. The Ad26.RSV.preF vaccine candidate was immunogenic with acceptable safety in a phase 1/2a study of RSV-seropositive children. Here, we assessed its safety and immunogenicity in RSV-seronegative children.

**Methods:**

In this randomized, observer-blinded, placebo-controlled, phase 1/2a study (NCT03606512; https://www.clinicaltrials.gov/ct2/show/NCT03606512), RSV-seronegative toddlers aged 12–24 months received Ad26.RSV.preF (2.5 × 10^10^ viral particles) or placebo on days 1, 29, and 57 (a meningococcal vaccine [Nimenrix] could substitute for day 57 placebo). Primary endpoints were solicited local and systemic adverse events (AEs; 7 days after each vaccination), unsolicited AEs (28 days postvaccination), and serious AEs (first vaccination until study end). Participants were monitored for RSV-respiratory tract infection to assess infection rates and for severe RSV-lower respiratory tract infection as an indication of enhanced disease. RSV-A2 neutralizing, RSV (A and B) preF binding, and RSV postF immunoglobulin G–binding antibodies were evaluated on days 1 (predose), 8, and 85, and after RSV season 1.

**Results:**

Thirty-eight participants were enrolled and vaccinated (Ad26.RSV.preF, n = 20; placebo, placebo/Nimenrix, n = 18). Solicited AEs were more common following Ad26.RSV.preF than placebo; most were mild/moderate. No vaccine-related serious AEs were reported. Five of 19 participants receiving Ad26.RSV.preF and 2/18 receiving placebo or placebo/Nimenrix had confirmed RSV-respiratory tract infection or RSV-associated otitis media; none were considered severe. At the final season 1 study visit, most Ad26.RSV.preF recipients had ≥2-fold increases from baseline in RSV-A2 neutralizing, RSV A and B preF binding, and RSV postF antibodies.

**Conclusions:**

Ad26.RSV.preF was well tolerated and immunogenic in RSV-seronegative toddlers.

## Teaser

In this phase 1/2a study, 3 doses of the Ad26.RSV.preF vaccine (2.5 × 10^10^ vp) was well tolerated and elicited an immunogenic response in RSV-seronegative toddlers aged 12–14 months through 2 RSV seasons.

## BACKGROUND

Respiratory syncytial virus (RSV) frequently causes severe respiratory illness in children [1, 2]. More than 80% of children aged 2 years have been exposed to RSV [3, 4]. Reinfection is common because of short-lived immunity conferred by natural infections [3, 5, 6].

Globally, ∼33 million RSV-associated lower respiratory tract infections (RSV-LRTIs) occur annually in children aged ≤5 years, resulting in 3.6 million hospitalizations and 101 400 deaths [7]. A recent study found that 1/56 healthy-term infants in high-income countries are hospitalized with RSV during the first year of life [8]; such hospitalizations require substantial medical resources [9]. Additionally, early-childhood RSV-LRTIs can be associated with later wheezing, asthma, and impaired lung function [10–12].

Historically, pediatric RSV vaccine development was hindered by vaccine-induced enhanced respiratory disease (ERD) that occurred in RSV-seronegative children infected with RSV after receiving a formalin-inactivated RSV vaccine in the 1960s [13, 14]. Subsequent studies suggested that ERD resulted from nonneutralizing humoral responses and Th2-skewed T-cell responses, prompting eosinophil and immune complex accumulation in the lungs after infection [13, 14]. A safe and effective RSV vaccine for children will likely need to induce potent neutralizing antibodies (nAbs) and Th1-skewed cellular immune responses, both of which protect against RSV [15, 16]. Antibodies against the RSV prefusion (preF) protein have the most potent neutralizing activity, whereas those against the RSV postfusion F (postF) protein have low neutralizing capability [17]. Recent progress has been made in developing passive immunization approaches, including maternal RSV vaccines and monoclonal antibodies, for RSV prophylaxis in children [18–20]. Nevertheless, development of a safe and efficacious vaccine for active immunization in young children remains an important need.

Ad26.RSV.preF is a replication-incompetent adenovirus serotype 26 (Ad26)–vectored vaccine encoding RSV preF protein stabilized in the prefusion conformation [21, 22]. In phase 1 and 2 adult trials, Ad26.RSV.preF elicited robust, lasting RSV-specific immune responses and was highly efficacious against RSV infection, with an acceptable safety profile [22–24].

In a phase 1/2a study evaluating immunogenicity and safety of 2 doses of Ad26.RSV.preF (5 × 10^10^ viral particles [vp]) in RSV-seropositive toddlers aged 12–24 months, the vaccine elicited RSV-A2 nAbs, RSV preF- and postF-specific antibodies, and predominantly Th1 cellular immune responses [25]. Although no safety concerns were raised, because of high rates of fever, the Ad26.RSV.preF dose for children was lowered to 2.5 × 10^10^ vp.

We evaluated safety, reactogenicity, and immunogenicity of Ad26.RSV.preF (2.5 × 10^10^ vp) in healthy RSV-seronegative toddlers aged 12–24 months.

## METHODS

### Study Design

This randomized, observer-blinded, multicenter, phase 1/2a study was conducted in Australia, Brazil, Canada, Finland, and Poland (ClinicalTrials.gov identifier: NCT03606512). The study initiated on 8 February 2019 and concluded on 2 November 2021, spanning 2 RSV seasons after dose 1, with most (25/38) participants randomized between 12 March 2019 to 22 November 2019. The beginning and end of the RSV seasons were determined using local or national surveillance data at study sites.

The study protocol was approved by the independent ethics committee/institutional review board at study sites. The trial was conducted in accordance with the Declaration of Helsinki and Good Clinical Practice. Parents or legally acceptable representatives of participants provided written informed consent before participation.

### Study Participants

Participants were screened within 6 weeks prior to vaccination. RSV-seronegative toddlers aged 12–24 months (inclusive) in good health without significant medical illness per physical examination, medical history, and vital signs at screening were eligible. RSV serostatus was assessed by RSV enzyme immunoassay (EIA) [3] at screening and confirmed by the absence of an anamnestic humoral immune response (a >4-fold increase in RSV A preF antibody enzyme-linked immunosorbent assay [ELISA]) at 7 days after dose 1. The Supplementary Materials provide additional eligibility criteria.

### Vaccination

Participants were centrally randomly assigned (1:1) to receive 3 doses of Ad26.RSV.preF (2.5 × 10^10^ vp; 0.25 mL) or placebo (0.25 mL) on days 1, 29, and 57. In countries where the meningococcal ACWY-tetanus toxoid vaccine Nimenrix (Pfizer) was licensed, placebo could be replaced by Nimenrix (0.5 mL) as the day 57 vaccination unless contraindicated (denoted as “placebo/Nimenrix”). Nimenrix was selected as control because its safety profile is comparable to that anticipated for Ad26.RSV.preF and to maintain blinding. The first 2 doses were planned for administration before the RSV season began. All doses were administered via intramuscular injection by a blinded administrator. Study dose rationale and first-dose safety methods are described in the Supplementary Materials.

### Study Objectives and Endpoints

The primary objective was to assess safety and reactogenicity of 3 doses of Ad26.RSV.preF (2.5 × 10^10^ vp) in healthy RSV-seronegative toddlers aged 12–24 months. Primary study endpoints were solicited local and systemic adverse events (AEs), unsolicited AEs, and serious AEs (SAEs).

Secondary endpoints included humoral immunogenicity (nAbs against RSV-A2, RSV A and B preF binding antibodies, and serum postF immunoglobulin G–binding antibodies) and occurrence of severe RSV-LRTI (severe LRTI by independent, blinded Clinical Endpoint Committee assessment with confirmation of RSV infection by reverse transcription polymerase chain reaction [RT-PCR] from a nasal sample). Blood was collected to assess cellular immune responses; these assessments were not reported because of low viable cell counts. Exploratory endpoints included nAbs to the Ad26 vector and RT-PCR–confirmed RSV-respiratory tract infection (RTI) and RSV-LRTI of any severity.

### Primary Endpoint Assessment

After each vaccination, participants were monitored for ≥30 minutes for acute reactions; after 30 minutes, vital signs, solicited local and systemic AEs, and unsolicited AEs were documented. Participants’ parents or legal guardians were provided a thermometer, ruler, and daily assessment diary to record solicited local and systemic AEs and body temperature. Solicited local and systemic AEs were recorded for 7 days, and unsolicited AEs were recorded for 28 days after each vaccination. SAEs were recorded from first dose until study end (ie, through 2 RSV seasons after dose 1). All AEs were followed until clinical resolution or stabilization.

### Secondary and Exploratory Endpoint Assessments

Blood samples for immunogenicity assessments were collected before dose 1 (or at screening), 7 days after dose 1 (day 8), 28 days after dose 3 (day 85), and at the end of the first RSV season. Immunogenicity assays (RSV-A2 neutralization assay, RSV A and B preF and RSV A postF binding antibody ELISAs, and Ad26 nAbs) were previously described [25]. Additional immunogenicity methods are described in the Supplementary Materials.

During the RSV season, participants’ parents or legal guardians recorded any signs or symptoms of RTI (eg, runny nose, fever, severe cough, wheezing, rapid breathing, difficulty breathing) or otitis media daily using a study-specific RTI symptoms form beginning at symptom onset until resolution. Outside the RSV season, participants’ parents or legal guardians recorded RTI signs and symptoms similarly for any medically attended RTIs. RTIs and otitis media cases were recorded through 2 RSV seasons after dose 1. Additional information is in the Supplementary Materials.

Participants’ parents or legal guardians were contacted by telephone or clinic visit through 2 RSV seasons after dose 1 (during RSV season: every 14 ± 3 days; outside RSV season: every 30 ± 7 days) and asked whether an RTI had occurred since the last call or visit and reminded to record any RTI or otitis media signs or symptoms; to contact study staff at symptom onset; and to record medically attended RTIs, concomitant medication use, and SAEs.

### Statistical Analysis

An original sample size of 24 participants per group was planned (total of 48 participants); however, this was amended to 18 participants per group (total of 36 participants) because of difficulties in recruiting seronegative toddlers. The full analysis set, which was used for safety analyses, included all randomly assigned participants who received ≥1 dose of study vaccine, regardless of protocol deviations.

The per-protocol immunogenicity (PPI) analysis set included all randomly assigned and vaccinated participants for whom immunogenicity data were available, excluding those with major protocol deviations anticipated to impact immunogenicity outcomes. Participants with an anamnestic humoral response (>4-fold increase in RSV A preF ELISA titers within 7 days after dose 1) were excluded from the PPI set. One participant who received Ad26.RSV.preF was excluded from the PPI set because of an anamnestic humoral response. If participants missed ≥1 dose but continued the study visit schedule, samples taken after the missed dose were excluded. Samples taken after an RT-PCR–confirmed RSV infection were excluded.

A modified intention-to-treat analysis set was defined as a subset of the full analysis set, excluding participants who were RSV-seronegative at screening but demonstrated an anamnestic humoral response. The modified intention-to-treat analysis set was used to assess RSV infection in RSV-seronegative participants.

The following analyses were conducted unblinded: primary analysis at 28 days after the final dose in all participants, including safety and immunogenicity; interim analysis when all participants had data for the first RSV season after dose 1, including safety and immunogenicity; and the final analysis at study completion.

Descriptive statistics (geometric mean titers [GMTs] and 95% confidence intervals [CIs]) were calculated for continuous immunologic parameters. No formal statistical testing was conducted. Safety data and the presence of RT-PCR–confirmed RSV infection were summarized descriptively.

## RESULTS

### Study Population

**Figure 1.**
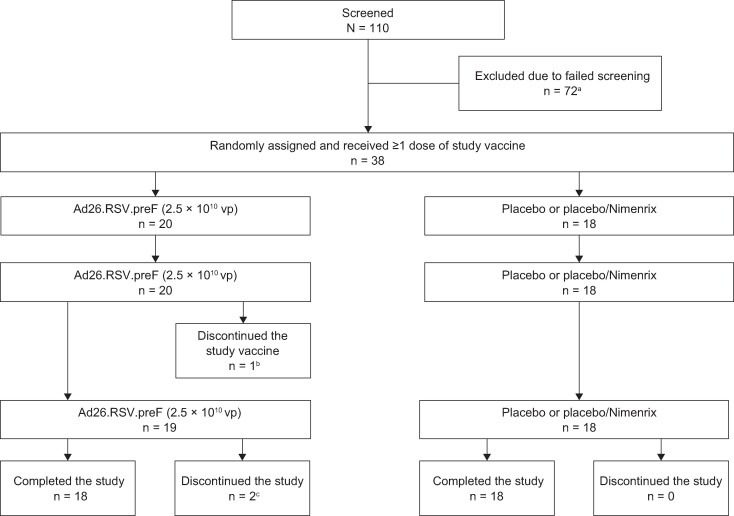
Participant disposition. RSV, respiratory syncytial virus; vp, viral particle. ^a^Seventy-two prospective participants had failed screenings, 59 because of RSV seropositivity. ^b^One participant discontinued the study vaccine after the second dose because of an unsolicited adverse event (urticaria) that was considered related to the study vaccine. ^c^One participant was lost to follow-up. The participant who discontinued the study vaccine because of an adverse event (urticaria) subsequently discontinued the study for other reasons (moved from study area).

Of 110 participants screened, 72 were excluded (59 of whom were seropositive). Thirty-eight participants were randomly assigned and vaccinated (Ad26.RSV.preF, n = 20; placebo or placebo/Nimenrix, n = 18; Figure 1), 37 completed the 3-dose regimen, and 36 completed the study. One participant in the Ad26.RSV.preF group discontinued the study vaccine after dose 2 because of an unsolicited AE (urticaria) that was considered related to study vaccine, and subsequently discontinued the study for other reasons (moved from the study area); another participant in this group was lost to follow-up after receiving all scheduled vaccinations.

Demographic characteristics were comparable across groups. Most participants were female (57.9%) and White (78.4%); the median (range) age was 16.5 (12–23) months (Table 1).

**Table 1.** Participant Demographics (Full Analysis Set)

Demographic	Ad26.RSV.preF/Ad26.RSV.preF/Ad26.RSV.preF (n = 20)	Placebo/placebo/placebo or Placebo/placebo/Nimenrix (n = 18)	All Participants (n = 38)
Sex, n (%)			
Female	11 (55.0)	11 (61.1)	22 (57.9)
Male	9 (45.0)	7 (38.9)	16 (42.1)
Age at screening, mo			
Median (range)	15.0 (12–23)	18.5 (12–22)	16.5 (12–23)
Race, n (%)	n = 19	n = 18	n = 37
White	16 (84.2)	13 (72.2)	29 (78.4)
Black/African American	2 (10.5)	1 (5.6)	3 (8.1)
Asian	1 (5.3)	1 (5.6)	2 (5.4)
Multiple	0	3 (16.7)	3 (8.1)
Ethnicity, n (%)			
Hispanic/Latino	3 (15.0)	2 (11.1)	5 (13.2)
Non-Hispanic/Latino	17 (85.0)	16 (88.9)	33 (86.8)
Length for age percentile, median (range)	48.5 (15.0–97.0)	50.0 (2.0–98.0)	50.0 (2.0–98.0)
Weight for age percentile, median (range)	79.4 (22.0–99.3)	59.5 (21.2–93.0)	68.6 (21.2–99.3)
Breastfeeding status, n (%)			
Did not breastfeed	2 (10.0)	1 (5.6)	3 (7.9)
Stopped breastfeeding	11 (55.0)	6 (33.3)	17 (44.7)
Currently breastfeeding	7 (35.0)	11 (61.1)	18 (47.4)
Family members’ smoking status, n (%)			
No smoker	18 (90.0)	16 (88.9)	34 (89.5)
Current smoker^a^	2 (10.0)	2 (11.1)	4 (10.5)

^a^Participants had ≥1 family member who smoked in the past or currently smokes.

### Safety

**Figure 2.**
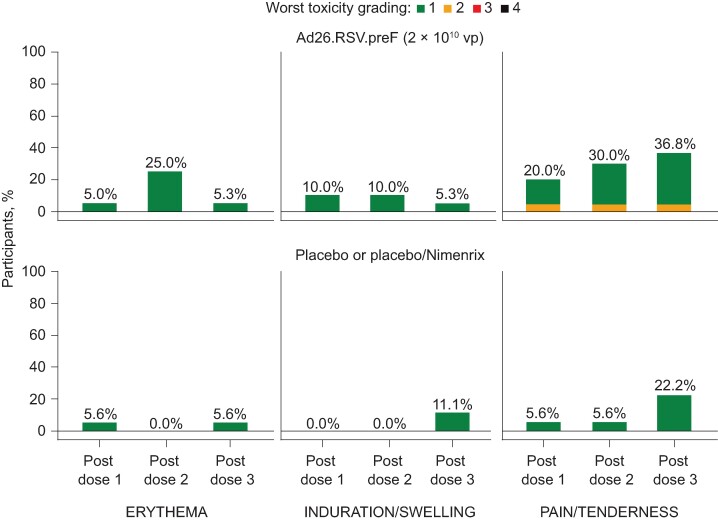
All solicited local AEs by worst severity grade after any vaccination (full analysis set). Ad26, adenovirus type 26 vector; AE, adverse event.

Solicited local AEs occurred within 7 days after any dose in 12/20 participants receiving Ad26.RSV.preF, 3/6 receiving placebo, and 4/12 receiving placebo/Nimenrix (Supplementary Table 1). All solicited local AEs were grade 1 or 2 in severity (Figure 2). The most common solicited local AE was injection site pain/tenderness, reported after any dose by 11/20 participants receiving Ad26.RSV.preF, 2/6 receiving placebo, and 3/12 receiving placebo/Nimenrix. Injection site erythema and swelling/induration were also more common following Ad26.RSV.preF (5/20 and 4/20, respectively) versus placebo (1/6 and 0/6) or placebo/Nimenrix (1/12 and 2/12). Solicited local AEs were generally transient, with a median duration of 1–4.5 days and median onset time of 1 day.

Solicited systemic AEs occurring within 7 days after any dose were reported in all (20/20) participants receiving Ad26.RSV.preF, 4/6 receiving placebo, and 9/12 receiving placebo/Nimenrix (Supplementary Table 2). The most common solicited systemic AE was irritability/crying (Ad26.RSV.preF: 18/20; placebo: 3/6; placebo/Nimenrix: 7/12). Decreased activity/lethargy (Ad26.RSV.preF: 12/20; placebo: 1/6; placebo/Nimenrix: 3/12), loss of appetite (9/20; 1/6; 4/12), fever (8/20; 0; 0), and diarrhea (6/20; 1/6; 3/12) were also more common following Ad26.RSV.preF than placebo or placebo/Nimenrix. Vomiting was slightly higher following placebo (2/6) than Ad26.RSV.preF or placebo/Nimenrix (4/20 and 1/12, respectively).

**Figure 3.**
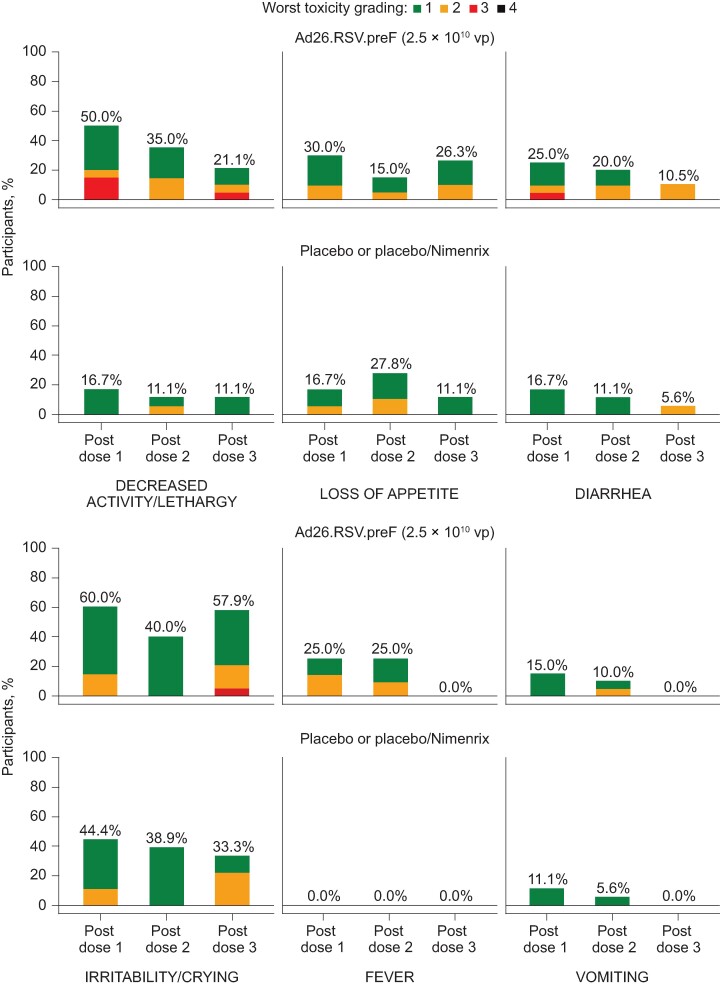
All solicited systemic AEs by worst severity grade after any vaccination (full analysis set). Ad26, adenovirus type 26 vector; AE, adverse event.

Most solicited systemic AEs were grade 1 or 2 in severity (Figure 3) and considered related to study vaccine by the investigator. Median onset time for solicited systemic AEs ranged from 1 to 4.5 days in Ad26.RSV.preF recipients and 1 to 7 days in placebo recipients, with median duration ranging from 1 to 4.5 days and 1 to 5.5 days, respectively. Grade 3 decreased activity/lethargy was reported for 3 participants after dose 1 and 1 participant after dose 3 in the Ad26.RSV.preF group. Grade 3 diarrhea was reported for 1 participant after dose 1 in the Ad26.RSV.preF group. Fever (body temperature ≥38°C) was observed in 5/20 participants after dose 1, 5/20 participants after dose 2, and 0/20 participants after dose 3 in the Ad26.RSV.preF group; no participants receiving placebo or placebo/Nimenrix reported fever. All fever events were grade 1 or 2, occurred on the day of vaccination, and resolved within 1 day.

Unsolicited AEs were reported in 6/20, 8/20, and 5/19 participants receiving Ad26.RSV.preF after dose 1, 2, and 3, respectively. Among placebo recipients, unsolicited AEs were reported in 2/18 participants after dose 1, 5/18 after dose 2, and 2/6 who received a third dose of placebo; among participants receiving Nimenrix as dose 3, 3/12 reported unsolicited AEs. All unsolicited AEs were grade 1 or 2, except for 1 instance of grade 3 diaper dermatitis in the Ad26.RSV.preF group, which was considered unrelated to study vaccine by the investigator. Most unsolicited AEs were considered unrelated to study vaccine by the investigator. The most common unsolicited AEs reported after any vaccination included RTI, upper RTI, and teething. One unsolicited grade 2 AE of urticaria that was considered related to study vaccine and resulted in study vaccine discontinuation occurred on the day of dose 2 (day 29) in the Ad26.RSV.preF group.

One SAE of sleep apnea syndrome occurred during follow-up in 1 participant in the Ad26.RSV.preF group, which was not considered related to study vaccine. No deaths were reported during the study.

Most participants (78.9%) received concomitant therapies, most commonly analgesics (eg, paracetamol, ibuprofen), in the 8 days after any dose of study vaccine (Supplementary Table 3).

### Immunogenicity

**Figure 4.**
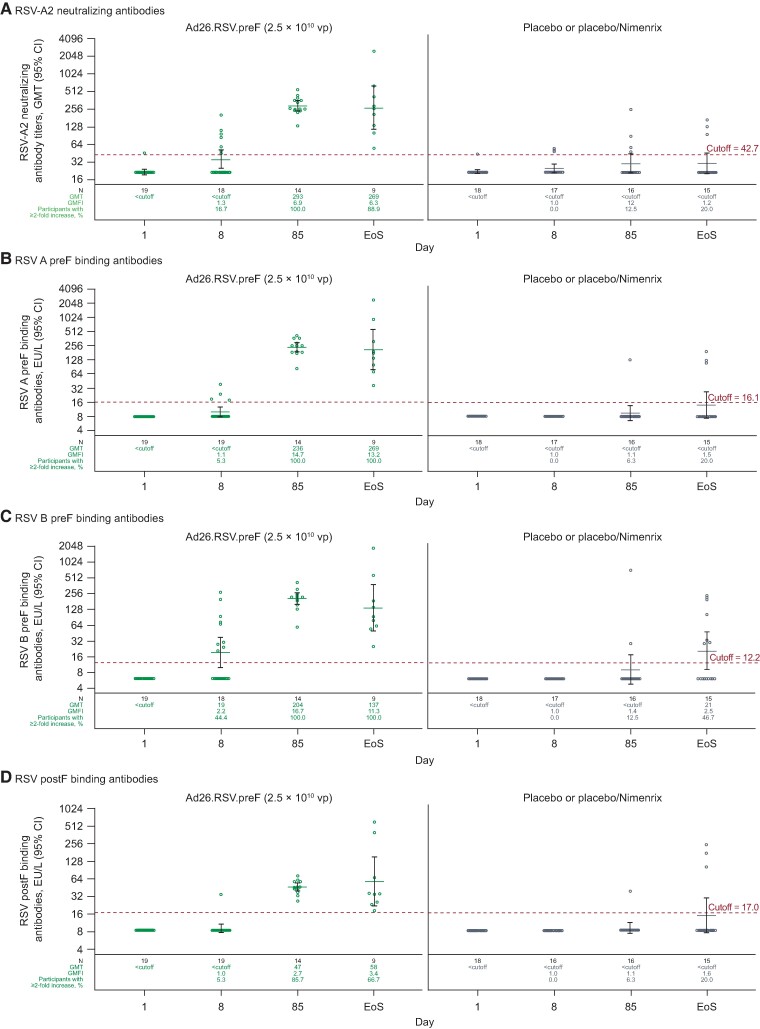
Titers of (*A*) RSV-A2 strain neutralizing antibodies and (*B*) RSV A preF, (*C*) RSV B preF, and (*D*) RSV postF binding antibodies (per-protocol immunogenicity analysis set). Ad26, adenovirus type 26 vector; ELISA, enzyme-linked immunosorbent assay; EoS, end of the first RSV season after study vaccination; EU, ELISA units; GMFI, geometric mean fold increase; GMT, geometric mean titer; postF, RSV postfusion conformation F protein; preF, RSV prefusion conformation F protein; RSV, respiratory syncytial virus; vp, viral particle.

The PPI analysis set comprised 19 participants receiving Ad26.RSV.preF and 18 receiving placebo or placebo/Nimenrix. Humoral immunogenicity data were missing primarily because of out-of-window visits and protocol deviations. At baseline, 1 participant in each of the Ad26.RSV.preF and placebo groups had titers above the virus neutralization assay seropositivity cutoff despite being seronegative at screening by EIA. At day 8, a different participant receiving Ad26.RSV.preF had a ≥4.0-fold increase from baseline in RSV-A2 nAbs; this participant was also seronegative at screening based on EIA (Figure 4*A*, Supplementary Figure 1A).

Of those with available data, 14/14 participants receiving Ad26.RSV.preF had a ≥2.0-fold increase from baseline in RSV-A2 nAbs (≥4.0-fold: 13/14) on day 85. At the end of the first RSV season, 8/9 participants had a ≥2.0-fold increase from baseline in RSV-A2 nAbs and 6/9 had a ≥4.0-fold increase. In the Ad26.RSV.preF group, GMTs for RSV-A2 nAbs were 293 (95% CI, 240–358) on day 85 and 269 (115–632) at the end of the first RSV season. In the placebo or placebo/Nimenrix group, GMTs were below the seropositivity threshold at all time points (Figure 4*A*, Supplementary Figure 1A). At the end of the first RSV season, 3/15 participants receiving placebo showed a ≥2.0-fold increase from baseline in RSV-A2 nAbs; none had a ≥4.0-fold increase.

At baseline, RSV A and B preF binding antibody titers were below the ELISA seropositivity cutoff for all participants. Similar to RSV-A2 nAb responses, at the end of the first RSV season, 9/9 participants receiving Ad26.RSV.preF with available data had ≥2.0-fold increases and 8/9 had ≥4.0-fold increases from baseline in RSV A (Figure 4*B*, Supplementary Figure 1B) and RSV B preF (Figure 4*C*, Supplementary Figure 1C) binding antibody titers. In the placebo and placebo/Nimenrix groups, GMTs of RSV A and B preF binding antibodies were below the seropositivity cutoff at all time points, except for GMTs of RSV B preF at the end of the RSV season (GMT [95% CI]: 21 [ <cutoff; 48] ELISA units/L).

Baseline RSV postF binding antibody titers were below the ELISA seropositivity cutoff in all groups (Figure 4*D*, Supplementary Figure 1D). At the end of the first RSV season, of those with available data, 6/9 participants receiving Ad26.RSV.preF had a ≥2.0-fold increase from baseline in postF binding antibody titers (≥4.0-fold: 2/9). In the placebo or placebo/Nimenrix group, GMTs of postF binding antibodies were below the seropositivity cutoff at all time points. At the end of the first RSV season, 3/15 participants receiving placebo or placebo/Nimenrix had a ≥4.0-fold increase from baseline in RSV postF antibody binding titers. Among Ad26.RSV.preF recipients, the ratios of fold increases in RSV postF binding antibodies to RSV-A2 nAbs were 0.8, 0.4, and 0.5 at day 8, day 85, and the end of the RSV season, respectively; corresponding ratios for placebo or placebo/Nimenrix recipients were 1.0, 0.9, and 1.3.

Preexisting Ad26 nAbs were only detected in 1 participant receiving placebo or placebo/Nimenrix; thus, impacts of baseline Ad26 seropositivity on immunogenicity were not assessed. On day 8, 13/19 participants receiving Ad26.RSV.preF had Ad26 nAb titers greater than the lower limit of quantification (LLOQ; GMT [95% CI]: 37 [21–66]) versus 2/17 receiving placebo or placebo/Nimenrix (GMT <LLOQ). On day 85, 14/14 participants receiving Ad26.RSV.preF with available data had Ad26 nAb titers >LLOQ (GMT [95% CI]: 956 [641–1425]) versus none receiving placebo or placebo/Nimenrix (GMT <LLOQ).

### RSV Infections

Among Ad26.RSV.preF recipients, 5/19 participants had RSV-RTI or RSV-associated otitis media, confirmed by RT-PCR. One participant had an RSV-RTI within the 28 days after dose 3, whereas 4 participants had an RSV-RTI during the follow-up phase (28 days after last received vaccination). No cases were medically attended.

In the placebo or placebo/Nimenrix group, 2/18 participants had RT-PCR–confirmed RSV-RTI or otitis media. One of these participants had a case of RSV-associated otitis media (not medically attended) following dose 3, and the other case was an RSV-RTI (medically attended) that occurred during follow-up. No RSV infections in either group were considered to be severe by the study investigators or assessed by the Clinical Endpoint Committee. Time-to-onset of first RSV infection is shown in Supplementary Figure 2, and RTI symptoms and grading are shown in Supplementary Table 4.

## DISCUSSION

Three doses of Ad26.RSV.preF (2.5 × 10^10^ vp) in healthy RSV-seronegative toddlers aged 12–24 months had an acceptable safety and tolerability profile. A lower dose was selected for this study to improve tolerability from the previous study of Ad26.RSV.preF (5 × 10^10^ vp) in RSV-seropositive toddlers [25]. Herein, pain/tenderness, decreased activity/lethargy, fever, and vomiting were more common following Ad26.RSV.preF (2.5 × 10^10^ vp) than placebo or placebo/Nimenrix; however, these events occurred less frequently than in the prior study of RSV-seropositive toddlers who were vaccinated with a higher dose. Most solicited local and systemic AEs were grade 1 or 2 in severity. Unsolicited AEs considered related to study vaccine were reported for 3 participants receiving Ad26.RSV.preF and 2 participants receiving placebo or placebo/Nimenrix. No SAEs occurred during the vaccination period; 1 SAE (sleep apnea syndrome) occurred in the Ad26.RSV.preF group during follow-up but was considered unrelated to the vaccine.

Notably, vaccine-induced immune thrombotic thrombocytopenia (VITT) has been observed at very low frequency following immunization with the Janssen Ad26.COV2.S COVID-19 vaccine. Because the mechanism for VITT is unconfirmed, it is unknown if VITT could occur with Ad26.RSV.preF. Because this study was conducted before identification of VITT with Ad26.COV2.S, thrombosis and thrombocytopenia events were not actively monitored. There was no evidence of thrombotic events in this study, although very few participants were vaccinated. Thrombotic events are to be monitored in all ongoing and future studies involving Ad26.RSV.preF.

Ad26.RSV.preF induced substantial increases in RSV-A2 nAbs and in RSV A and B preF and RSV postF binding antibodies in RSV-seronegative toddlers. Additionally, the ratio of fold increases in RSV postF binding antibodies to RSV-A2 nAbs ranged from 0.4 to 0.8 among Ad26.RSV.preF recipients across time points, which is a favorable observation given the association of vaccine-induced ERD with the induction of nonneutralizing postF-specific antibody response [13, 14]. Consistent with the lack of an immune response observed with placebo or placebo/Nimenrix, the ratio of fold increases in RSV postF binding antibodies to RSV-A2 nAbs in these groups was approximately 1 across time points (range, 0.9–1.3).

Because this study was not powered to evaluate vaccine efficacy, no conclusions regarding efficacy can be made. Furthermore, the study was conducted during the COVID-19 pandemic, potentially resulting in low RSV circulation during the study because of enacted public health measures [26]. Nevertheless, a greater proportion of participants in the Ad26.RSV.preF group versus the placebo group had confirmed RSV infection. Notably, none of the RSV infections were severe RSV-LRTIs, indicating no evidence of ERD. It is unclear whether the higher incidence of RSV infection among those in the Ad26.RSV.preF group occurred because of the overall low incidence of infection or whether the vaccine provides limited protection against mild RSV disease in this population of seronegative children. Further analyses are needed to determine immune correlates of protection against RSV infection.

This study had some limitations, including a smaller sample size than anticipated, in part because of a higher-than-expected seropositivity rate in the 12- to 14-month age group leading to difficulties in recruitment. Additionally, because of low numbers of viable cells obtained, we were unable to assess cellular immunogenicity, including Th-skewing, which would be important to ensure that the vaccine has a low risk of priming for ERD when administered to RSV-seronegative children. Importantly, data from studies of Ad26.RSV.preF-based vaccines in older adults and RSV-seropositive children, all of whom had RSV exposure, have shown that vaccine-induced cellular immune responses have a Th-1 signature [25, 27].

Very recently, nirsevimab and a maternal RSV vaccine were approved for prevention of RSV in young children and pregnant women, respectively [28, 29]. Although passive immunization of infants with RSV-specific monoclonal antibodies and maternal vaccines should not be overlooked [18, 19], active infant immunization offers potential advantages, including more durable protection and lower susceptibility to efficacy reductions resulting from F-protein mutations [30, 31]. Additionally, maternal vaccination must occur within a specific time frame, which may limit usefulness for vulnerable preterm infants [30]. Although the results presented here were encouraging, the development of the Ad26.RSV.preF vaccine was discontinued as the sponsor made a strategic decision to prioritize development of other assets. Ultimately, continued efforts are needed to develop an active prophylactic vaccine against RSV for children.

## Supplementary Material

ofae453_Supplementary_Data

## Supplementary Data

Supplementary materials are available at *Open Forum Infectious Diseases* online. Consisting of data provided by the authors to benefit the reader, the posted materials are not copyedited and are the sole responsibility of the authors, so questions or comments should be addressed to the corresponding author.

## Notes

***Acknowledgments.*** The authors thank all the investigators, participants and their families, and site personnel involved with this study. The authors thank Karin Weber for her contributions to the manuscript. Medical writing support was provided by William J. Kelley, PhD (Lumanity Communications Inc., Yardley, PA, USA).

***Author Contributions.*** J. M. L. contributed to study design/conceptualization, investigation, resources, project administration/supervision, and writing and reviewing/editing the manuscript. T. M. N. contributed to study design, study execution, data interpretation, and writing/editing the manuscript. M. R. contributed to data acquisition and writing/editing the manuscript. P. C. R. contributed to participant recruitment, data management, data acquisition, data analysis, and writing/editing the manuscript. N. R. F. contributed to data acquisition and editing the manuscript. W. H. contributed to study (medical) supervision, data (safety) analysis, and the reviewing/editing the manuscript. S. P. H. v. d. B. contributed to data acquisition, data analysis, data interpretation, and writing/editing the manuscript. K. W. contributed to trial design, startup activities, and reviewing/editing the manuscript. A. R. B. contributed to conceptualization, data interpretation (immunogenicity), sample analysis supervision and review, and reviewing/editing the manuscript. E. O. contributed to data collection, data analysis, data interpretation, and writing/editing the manuscript. J. W. D. contributed to data analysis and reviewing/editing the manuscript. N. S. contributed to conceptualization, data interpretation (immunogenicity), sample analysis supervision and review, and reviewing/editing the manuscript. G. V. G. contributed to data analysis and reviewing/editing the manuscript. W. v. D. contributed to formal analysis and writing/editing the manuscript. E. H. contributed to protocol design, conduct of study, data interpretation, and writing/reviewing the manuscript. B. C. contributed to study design, interpretation of results, and writing/editing the manuscript.

***Data availability.*** The data sharing policy of Janssen Pharmaceutical Companies of Johnson & Johnson is available at https://www.janssen.com/clinical-trials/transparency. As noted on this site, requests for access to the study data can be submitted through the Yale Open Data Access [YODA] Project site at http://yoda.yale.edu.

***Previous presentation.*** These results have not been previously presented or published.

***Financial support*.** This work was supported by Janssen Vaccines & Prevention B.V. during all stages of the trial and its analysis and through the development and publishing of the manuscript, including scientific writing assistance and statistical analyses.
