# Supplementary material for: A Phase 1/2a Study Evaluating Safety and Immunogenicity of Ad26.RSV.preF in RSV-seronegative Toddlers Aged 12–24 Months
Source: Open Forum Infect Dis. 2024 Aug 8;11(9):ofae453. doi: 10.1093/ofid/ofae453 (PMC11365064; doi:10.1093/ofid/ofae453)
Supplement: ofae453_Supplementary_Data [file ofae453_supplementary_data.docx]

**Supplementary Material**

**Supplementary Methods**

***Ethics Committees/Institutional Review Boards***

| **Country** | **Ethics committee/institutional review board** |
| --- | --- |
| Australia | Monash Health Human Research Ethics Committees |
|  | Research Ethics and Governance, The Royal Children's Hospital Melbourne |
|  | Barwon Health Human Research Ethics Committee, Research Governance Administrator Office for Research, Barwon Health |
|  | Women's and Children's Health Network Human Research Ethics Committee, WCHN |
|  | Telethon Kids Institute - Research Governance, Perth Children's Hospital |
| Brazil | Comite de Etica em Pesquisa do Hospital da Crianca Santo Antonio |
|  | National Committee of Ethics in Research (CONEP) |
|  | Comite de Etica em Pesquisa do Centro Universitario de Votuporanga - UNIFEV/SP |
|  | Comitê de Ética em Pesquisa do Hospital de Crianças César Perneta e Infantil Pequeno Príncipe |
|  | Comite de Etica em Pesquisa do Hospital das Clinicas da Faculdade de Medicina de Ribeirao Preto, Hospital das Clínicas da Faculdade de Medicina de Ribeirão Preto da Universidade de São Paulo - Divisão de Cardiologia |
|  | Comitê de Ética em Pesquisa do Hospital de Clínicas da Universidade Federal do Paraná |
|  | Comite de Etica em Pesquisa da Fundacao Jose Luiz Egydio Setubal |
|  | Comite de Etica em Pesquisa da PUCRS |
|  | Comite de Etica em Pesquisa da Universidade Federal do Espirito Santo |
|  | Comite de Etica em Pesquisa da Universidade Federal do Ceara |
| Canada | IWK Health Centre Research Ethics Board |
|  | Children's Hospital of Eastern Ontario Research Ethics Board |
|  | UBC Children's & Women’s Research Ethics Board |
|  | Conjoint Health Research Ethics Board (CHREB) Research Services, University of Calgary SMART Technologies Bldg |
|  | Comité d'éthique de la recherche du CHUQ |
|  | MUHC Center for Applied Ethics IRB/IEC |
| Finland | Hospital District of Southwest Finland Joint Municipal Authority Ethics Committee |
| Poland | Komisja Bioetyczna przy Dolnoslaskiej Izbie Lekarskiej we Wroclawiu |

***Eligibility Criteria***

*Inclusion criteria*

1. Each participants parent(s)/legal guardian(s) signed an informed consent form (ICF) indicating that he/she understands the purpose of and procedures required for the study, was willing for his/her child to participate in the study and attend all scheduled visits, and was willing and able to comply with all study procedures, including maintaining contact with the site for 2 RSV seasons following the first dose, and adhering to the prohibitions and restrictions specified.
2. Participant were male or female, whose age on the day of ICF signature was ≥12 months to ≤24 months and who were seronegative for RSV within 42 days prior to dosing.
3. Participant is the product of a normal term pregnancy ≥37 weeks, with a minimum birth weight of 2.5 kg.
4. Participant were in good health without any significant medical illness on the basis of physical examination, medical history, and vital signs performed at screening.
5. Participant had received all routine immunizations appropriate for his or her age according to local guidelines.
6. Each participant’s parent(s)/legal guardian(s) had access to a consistent means of contact either by telephone contact or email/computer.

*Exclusion criteria*

Any potential participant who met any of the following criteria were excluded from participating

in the study:

1. Participant had moderate or severe illness (this did not include minor illnesses such as diarrhea) or temperature ≥38.0 ºC within 24 hours prior to the first dose of study vaccine. In this situation, the participant was enrolled at a later date, or withdrawn at the discretion of the investigator and after consultation with the sponsor.
2. Any participant had an RTI between screening and randomization that the PI(s) felt made them ineligible.
3. Participant’s weight was below 10^th^ percentile according to World Health Organization (WHO) pediatric growth and weight charts.
4. Participant had any clinically significant acute or chronic medical condition (eg, history of seizure disorders, bleeding/clotting disorder, autoimmune disease, active malignancy, systemic infections, congenital heart disease, history of any pulmonary condition requiring medication, atopy, reactive airway disease, medically-confirmed wheezing, bronchoconstriction or treatment with a β2 agonist, cystic fibrosis, bronchopulmonary dysplasia, chronic pulmonary disease, medically-confirmed apnea, hospitalization for respiratory illness, or mechanical ventilation for respiratory illness) that, in the opinion of the investigator, precluded participation.
5. Participant had major congenital anomalies (after discussion with the SRP/S) or known cytogenetic disorders (eg, Down’s syndrome).
6. Participant had major surgery within the 4 weeks prior to randomization or had planned major surgery through the course of the study.
7. Participant was in receipt of, or planning to receive, live attenuated vaccine (eg, measles, mumps and rubella [MMR] or varicella, but excluding rotavirus vaccine) within 28 days of each study vaccination (ie, before and after); other vaccines (eg, influenza, pertussis, polio or rotavirus) were given at least 14 days before or 14 days after each study vaccination.
8. Participant had known or suspected immunodeficiency, such as known HIV infection.
9. Participant had received an investigational drug or used an invasive investigational medical device within 30 days or received an investigational vaccine within 6 months before the planned administration of the first dose of study vaccine or was currently enrolled or planned to participate in another investigational study during the study.
10. Participant had a known allergy to vaccines or vaccine components (including any of the constituents of the study vaccine), or history of anaphylaxis or other serious adverse reactions to vaccines or vaccine components (including any of the constituents of the study vaccine). Note that participant with egg allergies could be enrolled.
11. Participant had a history of the following moderate to severe chronic conditions: urticaria (recurrent hives), eczema and/or atopic dermatitis.
12. Participant had a history of acute polyneuropathy (eg, Guillain-Barré syndrome).
13. Participant had chronic or recurrent use of immunomodulators/suppressors, eg, cancer chemotherapeutic agents, oral or parenteral corticosteroids for at least 5 days within 42 days prior to randomization or planned during the study.
14. Participant had a history of receipt of blood products or immunoglobulin within 3 months of randomization.
15. Participant had been in receipt of palivizumab/Synagis® or received any other vaccine or monoclonal/polyclonal antibody in a previous RSV study at any time prior to randomization.
16. Participant had a contraindication to intramuscular injections and blood draws, eg, bleeding disorders.
17. Participant had a history of an underlying clinically significant acute or chronic medical condition or physical examination findings for which, in the opinion of the investigator, participation would not be in their best interest (eg, compromise the wellbeing) or that could prevent, limit, or confound the protocol-specified assessments.
18. Participant’s parent(s)/legal guardian(s) could not communicate reliably with the investigator.
19. Participant was a family member of either the investigator, an employee of the investigator or study site, with direct involvement in the proposed study or other studies under the direction of that investigator or study-site, or employee of the sponsor.

***Dose selection rationale***

In a phase 1/2a study (EudraCT Number: 2017-001345-27) evaluating immunogenicity and safety of 2 doses of Ad26.RSV.preF (5×10^10^ viral particles [vp]) or placebo in RSV-seropositive toddlers aged 12-24 months,^1^ an independent data monitoring committee (IDMC) reviewed interim analyses when 12 toddlers had reached Day 8 post-first dose. Their recommendation was that the study should continue unmodified. Transient fevers were observed in some participants, comparable in intensity to those seen following measles, mumps and rubella (MMR) immunization in this age group. Additionally, immunogenicity data from the study in 8 toddlers who received the first dose (5 after 5×10^10^ vp of Ad26.RSV.preF and 3 after placebo) showed that the vaccine was immunogenic, inducing a consistent increase in neutralizing antibody titers without affecting qualitative Th1/Th2 balances. Prior to dosing toddlers in the current study (ClinicalTrials.gov identifier: NCT03606512), the IDMC convened to review additional safety data (unblinded at the study group level) from the phase 1/2a study that had been collected up to that point (from at least the first 12 toddlers), including solicited adverse events (AEs), unsolicited AEs, and serious AEs (SAEs) through 28 days after the first dose of 5×10^10^ vp of Ad26.RSV.preF. The continued general safety and tolerability of the vaccine in the phase 1/2a study supported the expectation that safety would be acceptable at the dose of 2.5x10^10^ vp in the current study. Additional support for the 2.5×10^10^ vp dose used herein is the fact that this dose has been highly immunogenic for the individual Ad26 vector components in the sponsor’s HIV vaccine program where induction of Th1 responses in antigen-naïve individuals, similar to antigen-naïve RSV-seronegative toddlers, has been observed. It was therefore hypothesized that an adenovirus-vectored vaccine with a Th1 profile against RSV would reduce the likelihood of disease enhancement in RSV-seronegative recipients. Overall, the 2.5×10^10^ vp dose was thought to be an appropriate dose to start with for preliminary enhanced respiratory disease assessment in RSV-seronegative toddlers.

***First-dose Safety***

First-dose safety outcomes (solicited and unsolicited AEs and SAEs) were assessed after the first 8 participants received their first vaccination (Ad26.RSV.preF or placebo). An IDMC was in place throughout the study to monitor safety. Seven-day safety in these 8 participants was monitored by the Principal Investigator and the sponsor’s Study Responsible Physician. If no significant safety findings were reported, 4 additional participants were enrolled and vaccinated with Ad26.RSV.preF or placebo. The IDMC then reviewed reactogenicity data for 7 days post dose 1 for these 12 participants.

Second- and third-dose safety (solicited and unsolicited AEs and SAEs) was assessed by the Principal Investigator and the sponsor’s Study Responsible Physician at 7 days after the respective doses in the first 12 participants before vaccinating remaining participants.

***Assessments***

For this study, seropositivity cutoffs were 42.7 (half-maximum inhibitory concentration) for the RSV-A2 neutralization assay, 16.1 ELISA units (EU)/L for RSV A preF binding antibodies, 12.2 EU/L for RSV B preF binding antibodies, and 17.0 EU/L for RSV A postF binding antibodies. Ad26 nAbs were measured via virus neutralization assay with A549 cells and Ad26 expressing luciferase as a reporter gene and reported as 90% inhibitory concentration (IC_90_) values; an IC_90_ of 17 was the lower limit of quantification (LLOQ) in the assay.

Independent of RSV season, medically attended respiratory tract infections, including severe lower respiratory tract infections, and medically attended cases of otitis media, were reported. Medically attended was defined as healthcare professional visits to the home, clinic visits, emergency room attendance, or hospital admission sought by the participant’s parent/legal guardian or caregiver outside normal study procedures.

**References**

1. Stuart ASV, Virta M, Williams K, et al. Phase 1/2a Safety and Immunogenicity of an Adenovirus 26 Vector Respiratory Syncytial Virus (RSV) Vaccine Encoding Prefusion F in Adults 18-50 Years and RSV-Seropositive Children 12-24 Months. *J Infect Dis* 2022; **227**(1): 71-82.

**Supplementary Table 1. Solicited Local AEs (Full Analysis Set)**

| **Local adverse event** | **Ad26.RSV.preF/ Ad26.RSV.preF/ Ad26.RSV.preF** | **Placebo/ placebo/placebo** | **Placebo/ placebo/Nimenrix** |
| --- | --- | --- | --- |
| **Post-dose 1** | **Ad26.RSV.preF n=20** | **Placebo  n=18** | **-** |
| Solicited local AEs, n (%) |  |  |  |
| All grades | 6 (30.0) | 2 (11.1) | - |
| Grade 3/4 | 0 | 0 | - |
| Pain/tenderness |  |  |  |
| All grades | 4 (20.0) | 1 (5.6) | - |
| Grade 1/2 | 4 (20.0) | 0 | - |
| Erythema |  |  |  |
| All grades | 1 (5.0) | 1 (5.6) | - |
| Grade 1 | 1 (5.0) | 1 (5.6) | - |
| Induration/swelling |  |  |  |
| All grades | 2 (10.0) | 0 | - |
| Grade 1 | 2 (10.0) | 0 | - |
| **Post-dose 2** | **Ad26.RSV.preF n=20** | **Placebo**  **n=18** | **-** |
| Solicited local AEs, n (%) |  |  |  |
| All grades | 9 (45.0) | 1 (5.6) | - |
| Grade 3/4 | 0 | 0 | - |
| Pain/tenderness |  |  |  |
| All grades | 6 (30.0) | 1 (5.6) | - |
| Grade 1/2 | 6 (30.0) | 0 | - |
| Erythema |  |  |  |
| All grades | 5 (25.0) | 0 | - |
| Grade 1 | 5 (25.0) | 0 | - |
| Induration/swelling |  |  |  |
| All grades | 2 (10.0) | 0 | - |
| Grade 1 | 2 (10.0) | 0 | - |
| **Post-dose 3** | **Ad26.RSV.preF n=19** | **Placebo**  **n=6** | **Nimenrix**  **n=12** |
| Solicited local AEs, n (%) |  |  |  |
| All grades | 7 (36.8) | 1 (16.7) | 4 (33.3) |
| Grade 3/4 | 0 | 0 | 0 |
| Pain/tenderness |  |  |  |
| All grades | 7 (36.8) | 1 (16.7) | 3 (25.0) |
| Grade 1/2 | 7 (36.8) | 1 (16.7) | 3 (25.0) |
| Erythema |  |  |  |
| All grades | 1 (5.3) | 0 | 1 (8.3) |
| Grade 1/2 | 1 (5.3) | 0 | 1 (8.3) |
| Induration/swelling |  |  |  |
| All grades | 1 (5.3) | 0 | 2 (16.7) |
| Grade 1 | 1 (5.3) | 0 | 2 (16.7) |
| **Within 7 days post any dose** | **Ad26.RSV.preF/ Ad26.RSV.preF/ Ad26.RSV.preF**  **n=20** | **Placebo/ placebo/placebo**  **n=6** | **Placebo/ placebo/Nimenrix**  **n=12** |
| Solicited local AEs, n (%) | 12 (60.0) | 3 (50.0) | 4 (33.3) |

Ad26, adenovirus type 26 vector; AE, adverse event; preF, RSV prefusion conformation F protein; RSV, respiratory syncytial virus.

**Supplementary Table 2. Solicited Systemic AEs Occurring Within 7 Days of Study Vaccination (Full Analysis Set)**

| **Systemic adverse event** | **Ad26.RSV.preF/ Ad26.RSV.preF/ Ad26.RSV.preF** | **Placebo/ placebo/placebo** | **Placebo/ placebo/Nimenrix** |
| --- | --- | --- | --- |
| **Post-dose 1** | **Ad26.RSV.preF n=20** | **Placebo**  **n=18** | **-** |
| Any solicited systemic AE, n (%) | 17 (85.0) | 11 (61.1) | - |
| Grade 3 | 4 (20.0) | 0 | - |
| Irritability/crying | 12 (60.0) | 8 (44.4) | - |
| Decreased activity/lethargy | 10 (50.0) | 3 (16.7) | - |
| Loss of appetite | 6 (30.0) | 3 (16.7) | - |
| Diarrhea | 5 (25.0) | 3 (16.7) | - |
| Fever | 5 (25.0) | 0 | - |
| Vomiting | 3 (15.0) | 2 (11.1) | - |
| Solicited systemic AE related to study vaccine | 16 (80.0) | 10 (55.6) | - |
| **Post-dose 2** | **Ad26.RSV.preF n=20** | **Placebo**  **n=18** | **-** |
| Any solicited systemic AE, n (%) | 11 (55.0) | 9 (50.0) | - |
| Grade 3 | 0 | 0 | - |
| Irritability/crying | 8 (40.0) | 7 (38.9) | - |
| Decreased activity/lethargy | 7 (35.0) | 2 (11.1) | - |
| Loss of appetite | 3 (15.0) | 5 (27.8) | - |
| Diarrhea | 4 (20.0) | 2 (11.1) | - |
| Fever | 5 (25.0) | 0 | - |
| Vomiting | 2 (10.0) | 1 (5.6) | - |
| Solicited systemic AE related to study vaccine | 8 (40.0) | 8 (44.4) | - |
| **Post-dose 3** | **Ad26.RSV.preF n=19** | **Placebo**  **n=6** | **Nimenrix**  **n=12** |
| Any solicited systemic AE, n (%) | 12 (63.2) | 3 (50.0) | 4 (33.3) |
| Grade 3 | 2 (10.5) | 0 | 0 |
| Irritability/crying | 11 (57.9) | 2 (33.3) | 4 (33.3) |
| Decreased activity/lethargy | 4 (21.1) | 0 | 2 (16.7) |
| Loss of appetite | 5 (26.3) | 1 (16.7) | 1 (8.3) |
| Diarrhea | 2 (10.5) | 0 | 1 (8.3) |
| Fever | 0 | 0 | 0 |
| Vomiting | 0 | 0 | 0 |
| Solicited systemic AE related to study vaccine | 11 (57.9) | 3 (50.0) | 3 (25.0) |
| **Within 7 days post any dose** | **Ad26.RSV.preF/ Ad26.RSV.preF/ Ad26.RSV.preF**  **n=20** | **Placebo/ placebo/placebo**  **n=6** | **Placebo/ placebo/Nimenrix**  **n=12** |
| Solicited systemic AEs, n (%) | 20 (100) | 4 (66.7) | 9 (75.0) |

Ad26, adenovirus type 26 vector; AE, adverse event; preF, RSV prefusion conformation F protein; RSV, respiratory syncytial virus.

**Supplementary Table 3. Analgesics and Antipyretics Used by Study Participants Within 8 Days Post Any Dose of Study Vaccine**

| **Analgesics/antipyretics** | **Ad26.RSV.preF/ Ad26.RSV.preF/ Ad26.RSV.preF** | **Placebo/ placebo/placebo** | **Placebo/ placebo/Nimenrix** |
| --- | --- | --- | --- |
| **Post-dose 1** | **Ad26.RSV.preF n=20** | **Placebo  n=18** | **-** |
| Any analgesics/antipyretics, n (%) | 9 (45.0) | 1 (5.6) | - |
| Paracetamol | 6 (30.0) | 1 (5.6) |  |
| Ibuprofen | 6 (30.0) | 0 |  |
| **Post-dose 2** | **Ad26.RSV.preF n=20** | **Placebo  n=18** | **-** |
| Any analgesics/antipyretics, n (%) | 6 (30.0) | 0 | - |
| Paracetamol | 2 (10.0) | 0 | - |
| Ibuprofen | 4 (20.0) | 0 | - |
| Naproxen | 1 (5.0) | 0 | - |
| **Post-dose 3** | **Ad26.RSV.preF n=19** | **Placebo  n=6** | **Nimenrix**  **n=12** |
| Any analgesics/antipyretics, n (%) | 6 (31.6) | 1 (16.7) |  |
| Paracetamol | 5 (26.3) | 1 (16.7) | 1 (8.3) |
| Ibuprofen | 2 (10.5) | 0 | 1 (8.3) |
| Naproxen | 1 (5.3) | 0 | 0 |

Ad26, adenovirus type 26 vector; preF, RSV prefusion conformation F protein; RSV, respiratory syncytial virus.

**Supplementary Table 4. Summary of RTI Symptoms and Grading (Modified Intent-to-treat Analysis Set)**

| **Symptoms and Grading, n (%)** | **Ad26.RSV.preF/ Ad26.RSV.preF/ Ad26.RSV.preF**  **(n=19)** | **Placebo/ placebo/Nimenrix**  **(n=18)** |
| --- | --- | --- |
| Onsite form |  |  |
| Runny nose | 16 (84.2) | 16 (88.9) |
| Does not have this symptom | 0 | 2 (11.1) |
| Very mild | 2 (10.5) | 1 (5.6) |
| Mild | 8 (42.1) | 5 (27.8) |
| Moderate | 6 (31.6) | 6 (33.3) |
| Significant | 0 | 1 (5.6) |
| Severe | 0 | 1 (5.6) |
| Sneezing | 16 (84.2) | 16 (88.9) |
| Does not have this symptom | 7 (36.8) | 6 (33.3) |
| Very mild | 0 | 4 (22.2) |
| Mild | 8 (42.1) | 6 (33.3) |
| Moderate | 1 (5.3) | 0 |
| Congestion | 16 (84.2) | 16 (88.9) |
| Does not have this symptom | 1 (5.3) | 5 (27.8) |
| Very mild | 2 (10.5) | 0 |
| Mild | 9 (47.4) | 4 (22.2) |
| Moderate | 4 (21.1) | 7 (38.9) |
| Purulent Nasal Discharge (Yellow/Green) | 16 (84.2) | 16 (88.9) |
| Does not have this symptom | 6 (31.6) | 10 (55.6) |
| Very mild | 1 (5.3) | 2 (11.1) |
| Mild | 4 (21.1) | 3 (16.7) |
| Moderate | 5 (26.3) | 1 (5.6) |
| Cough | 16 (84.2) | 16 (88.9) |
| Does not have this symptom | 2 (10.5) | 5 (27.8) |
| Very mild | 2 (10.5) | 1 (5.6) |
| Mild | 6 (31.6) | 7 (38.9) |
| Moderate | 5 (26.3) | 2 (11.1) |
| Significant | 1 (5.3) | 1 (5.6) |
| Abnormal Breathing | 16 (84.2) | 16 (88.9) |
| Does not have this symptom | 14 (73.7) | 13 (72.2) |
| Very mild | 1 (5.3) | 1 (5.6) |
| Mild | 1 (5.3) | 2 (11.1) |
| Reported Lethargy | 16 (84.2) | 16 (88.9) |
| Does not have this symptom | 7 (36.8) | 8 (44.4) |
| Very mild | 1 (5.3) | 2 (11.1) |
| Mild | 5 (26.3) | 2 (11.1) |
| Moderate | 3 (15.8) | 3 (16.7) |
| Significant | 0 | 1 (5.6) |
| Reported Decreased Appetite | 16 (84.2) | 16 (88.9) |
| Does not have this symptom | 11 (57.9) | 8 (44.4) |
| Mild | 5 (26.3) | 3 (16.7) |
| Moderate | 0 | 5 (27.8) |
| Chills | 16 (84.2) | 16 (88.9) |
| Does not have this symptom | 14 (73.7) | 13 (72.2) |
| Very mild | 1 (5.3) | 2 (11.1) |
| Moderate | 1 (5.3) | 1 (5.6) |
| Irritability/Crying | 16 (84.2) | 16 (88.9) |
| Does not have this symptom | 7 (36.8) | 6 (33.3) |
| Very mild | 0 | 2 (11.1) |
| Mild | 5 (26.3) | 4 (22.2) |
| Moderate | 4 (21.1) | 3 (16.7) |
| Significant | 0 | 1 (5.6) |
| Child Taken To A Doctor | 16 (84.2) | 16 (88.9) |
| No | 13 (68.4) | 10 (55.6) |
| Yes | 3 (15.8) | 6 (33.3) |
| Child Taken To Hospital | 16 (84.2) | 16 (88.9) |
| No | 14 (73.7) | 14 (77.8) |
| Yes | 2 (10.5) | 2 (11.1) |
| Otitis Media | 16 (84.2) | 16 (88.9) |
| No | 15 (78.9) | 11 (61.1) |
| Yes | 1 (5.3) | 5 (27.8) |
| Home form |  |  |
| How Sick Has Child Felt In Last 24 Hrs? | 15 (78.9) | 16 (88.9) |
| Mildly sick | 3 (15.8) | 2 (11.1) |
| Moderately sick | 10 (52.6) | 3 (16.7) |
| Very sick | 2 (10.5) | 7 (38.9) |
| Runny nose | 15 (78.9) | 16 (88.9) |
| Very mild | 1 (5.3) | 2 (11.1) |
| Mild | 2 (10.5) | 1 (5.6) |
| Moderate | 6 (31.6) | 4 (22.2) |
| Significant | 4 (21.1) | 5 (27.8) |
| Severe | 2 (10.5) | 1 (5.6) |
| Sneezing | 15 (78.9) | 16 (88.9) |
| Does not have this symptom | 1 (5.3) | 3 (16.7) |
| Very mild | 3 (15.8) | 4 (22.2) |
| Mild | 4 (21.1) | 5 (27.8) |
| Moderate | 6 (31.6) | 3 (16.7) |
| Significant | 1 (5.3) | 1 (5.6) |
| Congestion | 15 (78.9) | 16 (88.9) |
| Very mild | 1 (5.3) | 1 (5.6) |
| Mild | 3 (15.8) | 2 (11.1) |
| Moderate | 4 (21.1) | 7 (38.9) |
| Significant | 7 (36.8) | 3 (16.7) |
| Purulent Nasal Discharge (Yellow/Green) | 15 (78.9) | 16 (88.9) |
| Does not have this symptom | 1 (5.3) | 3 (16.7) |
| Very mild | 0 | 2 (11.1) |
| Mild | 3 (15.8) | 3 (16.7) |
| Moderate | 5 (26.3) | 2 (11.1) |
| Significant | 6 (31.6) | 6 (33.3) |
| Child Taken To A Doctor | 15 (78.9) | 16 (88.9) |
| No | 9 (47.4) | 7 (38.9) |
| Yes | 6 (31.6) | 9 (50.0) |
| Child Taken To Hospital | 15 (78.9) | 16 (88.9) |
| No | 13 (68.4) | 15 (83.3) |
| Yes | 2 (10.5) | 1 (5.6) |
| Breathing More Rapidly | 15 (78.9) | 16 (88.9) |
| Does not have this symptom | 6 (31.6) | 8 (44.4) |
| Very mild | 2 (10.5) | 2 (11.1) |
| Mild | 3 (15.8) | 2 (11.1) |
| Moderate | 2 (10.5) | 4 (22.2) |
| Feeling Feverish | 15 (78.9) | 16 (88.9) |
| Does not have this symptom | 6 (31.6) | 6 (33.3) |
| Very mild | 0 | 2 (11.1) |
| Mild | 2 (10.5) | 2 (11.1) |
| Moderate | 6 (31.6) | 1 (5.6) |
| Significant | 1 (5.3) | 5 (27.8) |
| Shivering | 15 (78.9) | 16 (88.9) |
| Does not have this symptom | 11 (57.9) | 13 (72.2) |
| Very mild | 2 (10.5) | 1 (5.6) |
| Mild | 1 (5.3) | 1 (5.6) |
| Moderate | 1 (5.3) | 1 (5.6) |
| Ear Infection | 15 (78.9) | 16 (88.9) |
| Does not have this symptom | 14 (73.7) | 13 (72.2) |
| Mild | 0 | 1 (5.6) |
| Moderate | 1 (5.3) | 0 |
| Chest pain | 15 (78.9) | 16 (88.9) |
| Does not have this symptom | 14 (73.7) | 16 (88.9) |
| Moderate | 1 (5.3) | 0 |
| Headache | 15 (78.9) | 16 (88.9) |
| Does not have this symptom | 14 (73.7) | 16 (88.9) |
| Moderate | 1 (5.3) | 0 |
| Sore Throat | 15 (78.9) | 16 (88.9) |
| Does not have this symptom | 11 (57.9) | 14 (77.8) |
| Mild | 1 (5.3) | 1 (5.6) |
| Moderate | 2 (10.5) | 1 (5.6) |
| Severe | 1 (5.3) | 0 |

Ad26, adenovirus type 26 vector; preF, RSV prefusion conformation F protein; RSV, respiratory syncytial virus; RTI, respiratory tract infection.

**Supplementary Figure 1. Individual Participant Profiles for Titers of (A) RSV-A2 Strain Neutralizing Antibodies and (B) RSV A preF, (C) RSV B preF, and (D) RSV A postF Binding Antibodies (Per-protocol Immunogenicity Analysis Set)**


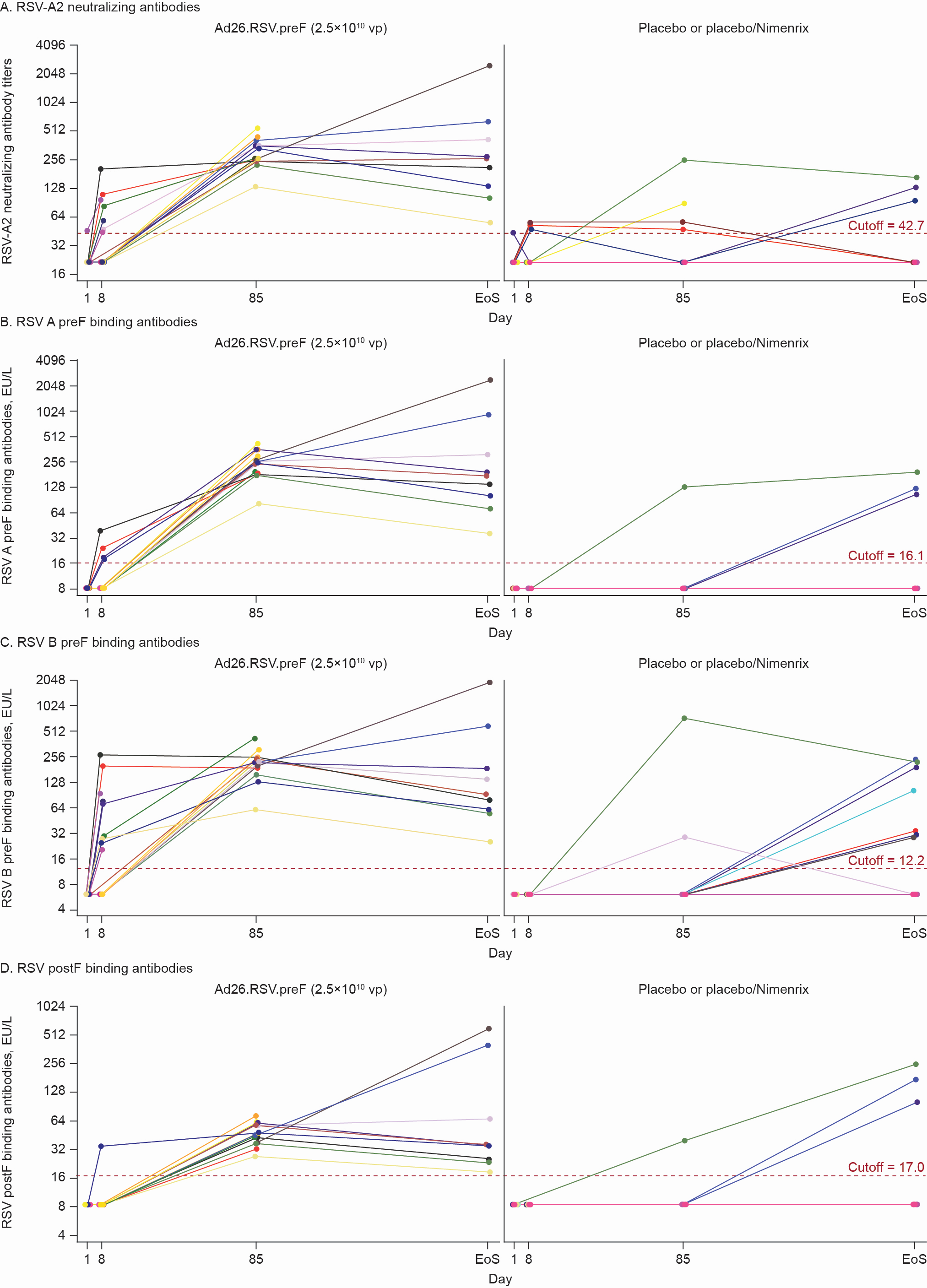


Ad26, adenovirus type 26 vector; ELISA, enzyme-linked immunosorbent assay; EoS, end of the first RSV season after study vaccination; EU, ELISA units; postF, RSV postfusion conformation F protein; preF, RSV prefusion conformation F protein; RSV, respiratory syncytial virus; vp, viral particle.

**Supplementary Figure 2. Time-to-onset of First RSV Infection (Modified Intent-to-treat Analysis Set)**


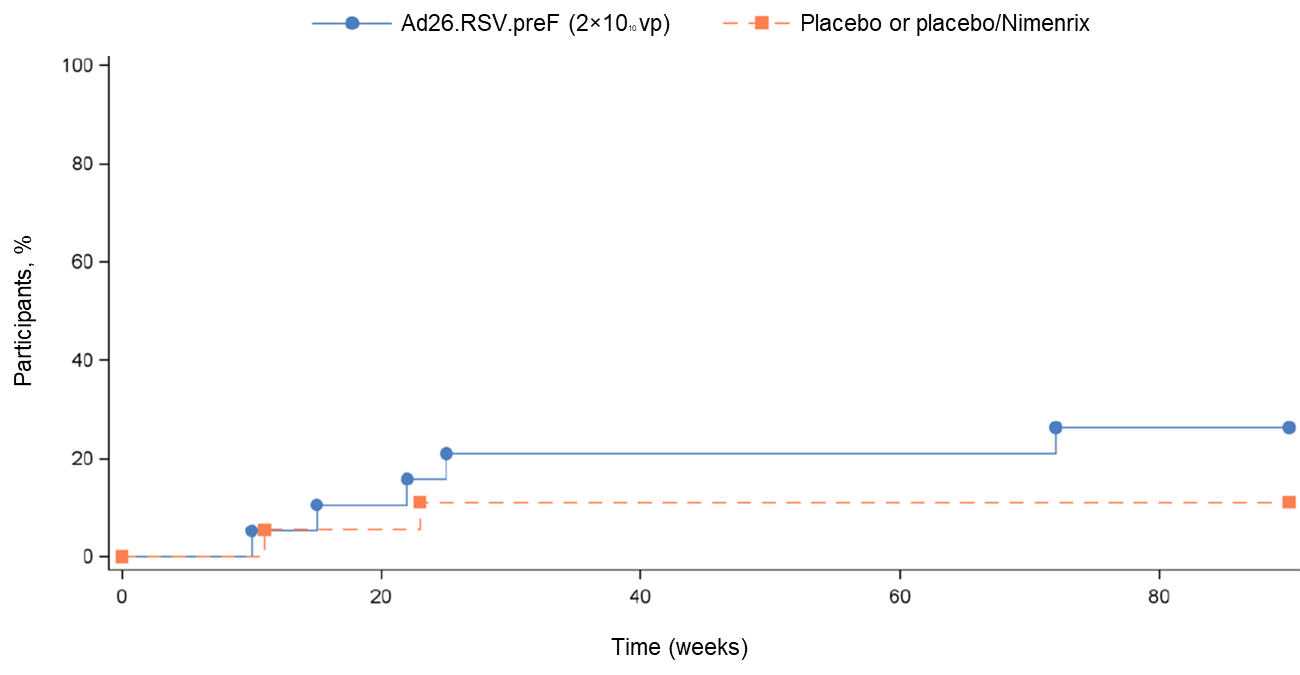


Ad26, adenovirus type 26 vector; preF, RSV prefusion conformation F protein; RSV, respiratory syncytial virus; vp, viral particle.
